# The complete chloroplast genome sequence of *Styrax odoratissimus* (Styracaceae)

**DOI:** 10.1080/23802359.2019.1688705

**Published:** 2019-11-14

**Authors:** Yaoqin Zhang, Lili Tong, Xiaogang Xu, Zixun Zhao, Yabo Wang

**Affiliations:** a College of Biology and Environment, Nanjing Forestry University, Nanjing, China;; b Co-Innovation Center for Sustainable Forestry in Southern China, Nanjing Forestry University, Nanjing, China;; c School of Horticulture and Landscape Architecture, Jinling Institute of Technology, Nanjing, China

**Keywords:** *Styrax odoratissimus*, Styracaceae, complete chloroplast genome, phylogenetic analysis

## Abstract

*Styrax odoratissimus* Champ. ex Benth., also known as the extraordinary aromatic plant of Styracaceae, has been seriously threatened due to human activities. In this study, the complete chloroplast (cp) genome sequence of *S. odoratissimus* was determined by applying next-generation sequencing. The entire cp genome was determined to be 157,921 bp in length. It contained large single-copy (LSC) and small single-copy (SSC) regions of 87,524 bp and 18,301 bp, respectively, which were separated by a pair of 26,048 bp inverted repeat (IR) regions. The genome contained 130 genes, including 85 protein-coding genes, 37 tRNA genes, and 8 rRNA genes. The overall GC content of *S. odoratissimus* genome is 36.96% and the corresponding values in LSC, SSC, and IR regions are 34.80, 30.27, and 42.92%, respectively. A phylogenetic tree reconstructed by 31 chloroplast genomes reveals that *S. odoratissimus* is most related to *Styrax grandiflorus* Griffith.

*Styrax odoratissimus* Champ. ex Benth., named after its pleasant fragrance of the flowers, is an endemic species to China in *Styrax* (Huang and James 2003). It can be as applied for ornamental, aromatic, pharmaceutical purposes. However, its wild resources have been seriously threatened with deforestation. In addition, its phylogenetic position is unclear in previous studies because of the lack of genomic resources of *Styrax* genus. Chloroplast genomes are widely applied to studies of species conservation, genome evolution, and phylogeny (Jansen et al. 2007; Moore et al. 2007). Consequently, the genetic and genomic information is urgently needed to promote its systematic research and the development of conservation value of *S. odoratissimus*. In this study, we characterized the complete chloroplast (cp) genome sequence of *S. odoratissimus* (GeneBank accession number: MN368610) based on Illumina pair-end sequencing to provide a valuable complete cp genomic resource.

In this research work, the fresh leaves of *S. odoratissimus* were collected from Shimenxia (N 30.08, E 118.14) of Huangshan Mountain Area, Anhui Province, China. The voucher specimen was deposited in the herbarium of Nanjing Forestry University (accession number: NF2018038). The total DNA extracting and the whole-genome sequencing were conducted by Nanjing Genepioneer Biotechnologies Inc. (Nanjing, China) with the Illumina Hiseq 2500 Sequencing System. The raw reads were filtered by CLC Genomics Workbench v9, and filtered sequences were assembled using the program SPAdes assembler v3.10.1 (Bankevich et al. 2012). Finally, gene structure annotation was carried out with CpGAVAS (Liu et al. 2012) and the physical map was generated with OGDRAW (Lohse et al. 2013).

The circular genome of *S. odoratissimus* was 157,921 bp in size and contained two inverted repeat (IRa and IRb) regions of 26,048 bp, which were separated by a large single-copy (LSC) region of 87,524 bp, and a small single-copy (SSC) region of 18,301 bp. The genome contained 130 genes, including 85 protein-coding genes, 37 tRNA genes, and 8 rRNA genes. Six protein-coding genes *(ndhB, rpl2, rpl23, rps7, ycf15, and ycf2*), seven tRNA genes (*tRNA-ACG*, *tRNA-CAA*, *tRNA-CAU*, *tRNA-GAC*, *tRNA-GUU*, *tRNA-UGC*, *tRNA-UUC*), and four rRNA genes are duplicated in the IR regions. A total of five protein-coding genes (*atpF*, *ndhB*, *rpl2*, *rpoC1*, and *rps16*) contained one intron while the other one gene (*clpP*) had two introns each. The overall GC content of *S. odoratissimus* genome is 36.96% and the corresponding values in LSC, SSC, and IR regions are 34.80, 30.27, and 42.92%, respectively.

To figure out its taxonomic status, alignment was performed on the 31 chloroplast genome (19 chloroplast genomes of Styracaceae and 5 taxa as outgroup) sequences using MAFFT v7.307 (Katoh and Standley 2014), and a maximum likelihood (ML) was manually modified using Figtree v1.4.4. The ML phylogenetic tree shows that *S. odoratissimus* is most related to *Styrax grandiflorus*, with bootstrap support values of 100% (Figure 1).

**Figure 1.**
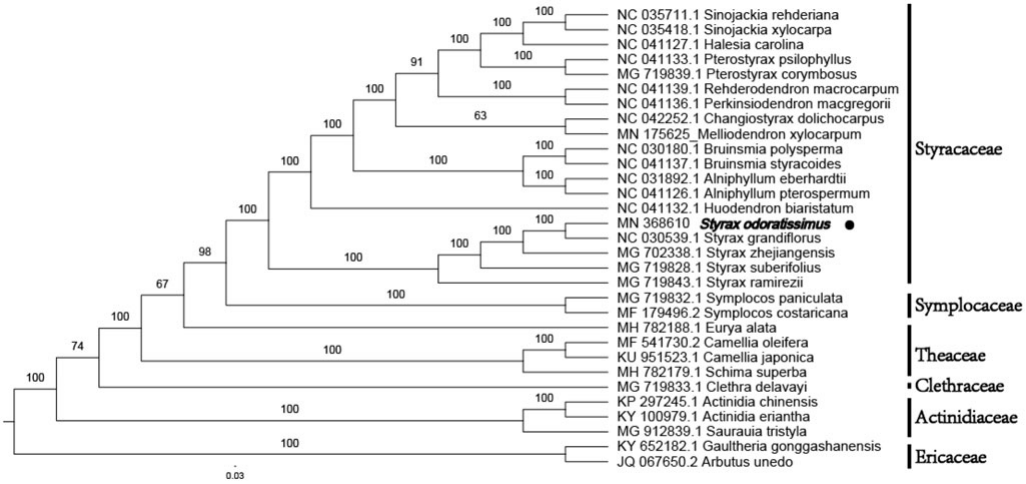
Phylogenetic tree inferred by maximum-likelihood (ML) method based on the complete chloroplast genome of 31 representative species. The bootstrap support values are shown at the branches.

## References

[CIT0001] Bankevich A, Nurk S, Antipov D, Gurevich AA, Dvorkin M, Kulikov AS, Lesin VM, Nikolenko SI, Pham S, Prjibelski AD, et al. 2012 SPAdes: a new genome assembly algorithm and its applications to single-cell sequencing. J Comput Biol. 19(5):455–477. 22506599 10.1089/cmb.2012.0021 PMC3342519

[CIT0002] Huang S-M, James WG 2003 Styracaceae In: Wu Z-Y, Raven PH, Hong D-Y, editors. Flora of China (Styracaceae). Vol. 15 Beijing: Science Press/St. Louis: Missouri Botanic Garden Press; p. 258.

[CIT0003] Jansen RK, Cai Z, Raubeson LA, Daniell H, dePamphilis CW, Leebens-Mack J, Muller KF, Guisinger-Bellian M, Haberle RC, Hansen AK, et al. 2007 Analysis of 81 genes from 64 plastid genomes resolves relationships in angiosperms and identifies genome-scale evolutionary patterns. Proc Natl Acad Sci USA. 104(49):19369–19374. 18048330 10.1073/pnas.0709121104 PMC2148296

[CIT0004] Katoh K, Standley DM 2014 MAFFT: iterative refinement and additional methods. Methods Mol Biol. 1079:131–146. 24170399 10.1007/978-1-62703-646-7_8

[CIT0005] Liu C, Shi LC, Zhu YJ, Chen HM, Zhang JH, Lin XH, Guan XJ 2012 CpGAVAS, an integrated web server for the annotation, visualization, analysis, and GenBank submission of completely sequenced chloroplast genome sequences. BMC Genomics. 13(1):715. 23256920 10.1186/1471-2164-13-715 PMC3543216

[CIT0006] Lohse M, Drechsel O, Kahlau S, Bock R 2013 Organellar Genome DRAW—a suite of tools for generating physical maps of plastid and mitochondrial genomes and visualizing expression data sets. Nucleic Acids Res. 41(W1):W575–W581. 23609545 10.1093/nar/gkt289 PMC3692101

[CIT0007] Moore MJ, Bell CD, Soltis PS, Soltis DE 2007 Using plastid genome-scale data to resolve enigmatic relationships among basal angiosperms. Proc Natl Acad Sci USA. 104(49):19363–19368. 18048334 10.1073/pnas.0708072104 PMC2148295

